# Transcranial Doppler Ultrasound for Monitoring the Cerebral Hemodynamic Changes and Prognosticating Outcomes in Venoarterial Extracorporeal Membrane-Oxygenated Patients

**DOI:** 10.1155/2022/2912477

**Published:** 2022-08-22

**Authors:** Man Wang, Le Li, Yi-Dong Tan

**Affiliations:** Emergency Department, Guigang City People's Hospital, Guigang 537100, Guangxi Zhuang Autonomous Region, China

## Abstract

**Objective:**

Patients receiving venoarterial extracorporeal membrane oxygenation (VA-ECMO) support may have cerebral hemodynamic changes whose impact on patient outcome are not fully elucidated. This study aims to evaluate the correlation between cerebral hemodynamic changes and prognostic outcome in patients during VA-ECMO.

**Methods:**

Transcranial Doppler (TCD) ultrasound examination was performed to attain the systolic velocity (Vs), diastolic velocity (Vd), mean velocity (Vm), and pulsatility index (PI) of patients undergoing VA-ECMO. Cardiac ultrasound was also performed to assess the correlation between the left ventricular outflow tract velocity time integral (LVOT VTI), left ventricular ejection fraction (LVEF), and middle cerebral artery (MCA) with the systolic peak. Moreover, we assessed the predictive value of LVOT VTI and LVEF in patients with the systolic peak. Patients were divided into survival and death groups according to the 28-day survival period. Clinical data were compared between the two groups to investigate the effects of cerebral hemodynamic changes on the prognosis of VA-ECMO patients.

**Results:**

We found that the patient's LVOT VTI and LVEF had high predictive values for the systolic peak of the right middle cerebral artery. The initial LVEF, Vs, Vd and PI, and lactate level as well as the MODS incidence rate difference were significantly different between the survival and death groups. In addition, the results showed that the initial Vs value was an independent risk factor for the prognosis of patients undergoing VA-ECMO.

**Conclusions:**

Cerebral hemodynamic changes may occur in patients supported by VA-ECMO. In addition, a poor cerebral arterial pulsatile blood flow was closely correlated with an unfavorable outcome in these patients.

## 1. Introduction

The most common complication in patients undergoing venoarterial extracorporeal membrane oxygenation (VA-ECMO) is brain injury. It occurs in 8–50% of VA-ECMO patients [1–5], and brain death occurs in 7–21% of them [6]. The increasing trend of neurological complications over time may reflect a degree of under-reporting in earlier studies and an increased awareness over time. However, inadequate assessment and detection of this condition have contributed to underestimation of neurological complications [5, 7]. Due to limited clinical examination choices and the uncertainty of the effective neurologic monitoring methods, little is known about the cerebral hemodynamic changes during VA-ECMO. Consequently, the monitoring of cerebral hemodynamic changes in patients with VA-ECMO by easily implemented methods is of great concern in clinical practice.

Transcranial Doppler (TCD) ultrasound is an important part of multimodal brain monitoring that is normally performed in two main ways: traditional nonimaging TCD and transcranial color code sonography (TCCS). It is a noninvasive ultrasound technique that uses a low-frequency transducer probe to insonate the cerebral arteries through relatively thin bone windows [8]. TCD is widely used in the assessment of acute ischemic stroke [9], cerebral aneurysm [10], sickle cell anemia [11], senile dementia [12, 13], migraine [14], and other diseases. In recent years, TCD has been increasingly applied to monitor the cerebral autoregulation that occurs in coma patients [15, 16]. TCD provides visual technical support for monitoring brain functions in VA-ECMO patients, and it has the advantages of being noninvasive. It can also be used at the bedside and is easily operated and repeatable without the risk of radiation. It is now considered to be an important tool for evaluating the cerebrovascular status and cerebral hemodynamics of patients. At present, the use of TCD based in ECMO patients is still in the exploratory stage. Previous clinical studies based on TCD changes in VA-ECMO patients were observed in small samples, and no strong statistical analysis has been performed. In addition, the effects of the TCD pattern changes in the patients' prognosis have not been clarified.

Clinically, we found that nonpulsatile circulation supported by VA-ECMO can significantly change a patient's TCD waveform and the cerebral hemodynamic changes seen can have close correlation to cardiac function. Therefore, we suggest that cardiac systolic function can be used as a predictor of changes in the TCD patterns, and the changes in cerebral hemodynamics may be associated with disease severity and prognosis. This study combined the use of TCD ultrasound in VA-ECMO patients with clinical data and aimed to observe the changes of cerebral hemodynamic in different cardiac function states. This allowed us to explore the correlation between cerebral hemodynamic changes and prognosis by using comparison and regression analyses between the survival and death groups.

## 2. Materials and Methods

### 2.1. Study Subject and Clinical Resources

This study was performed according to the approval of the Ethics Committee Board of the Guigang People's Hospital (ethics approval reference number: GYLLPJ-20210511-03), and all study activities conformed to the principles expressed in the Declaration of Helsinki for studies on human subjects. Written informed consent was obtained from all subjects prior to participation in this study.

The VA-ECMO patients admitted to emergency intensive care unit (EICU) of Guigang People's Hospital from January 2020 to January 2022 were included in this study. The inclusion criteria were as follows: age ≥18 years and patients with VA-ECMO support. The exclusion criteria were as follows: patients with TCDs without any acoustic window (i.e., a cerebral blood flow signal could not be obtained); a primary cerebral injury (ischemic or hemorrhagic cerebrovascular events, intracranial tumor, and intracranial infection); severe carotid artery or intracranial artery stenosis (>70%); and premorbid cognitive disorders such as dementia.

### 2.2. VA-ECMO Establishment and Management

Professionally trained ECMO team members cannulated each patient for VA-ECMO via the femoral vein and artery. 64 patients were cannulated via a percutaneous approach guided by visual ultrasound, and 2 patients were cannulated with surgical venous cutdown. An 8Fr arterial catheter was placed routinely in the superficial femoral artery of the ipsilateral lower extremity to improve perfusion. The ECMO circuit consisted of a membrane oxygenator (PLS-i 2050, Maquet, Germany), centrifugal pump (Rotaflow, Maquet, Germany), arteriovenous cannula (BE-PVL/PVS/PAL, Maquet, Germany), heparin-coated circuit, an air-oxygen mixer, and a variable temperature water tank. The ECMO flow was adjusted to provide systemic perfusion, and indicators such as the mixed venous oxygen saturation and the blood lactate levels were monitored to assess perfusion. Individualized anticoagulation was performed by monitoring the patients' routine coagulation and thromboelastography-related indicators. None of the patients used other cardiac-assisted devices during this time period.

### 2.3. TCD Ultrasound Examination

Professionally trained team members performed the TCD ultrasound examinations for all patients during the first 24 hours from the start of ECMO. The machine used for performing TCDs was a M9 portable color Doppler ultrasound system (Minray Biomedical Electronics Co., Ltd., Shenzhen, China) normally used for bedside evaluations. A low-frequency (1.5–3 MHz) probe was selected to measure the blood flow of the right middle cerebral artery (RMCA) through the temporal window in order to observe whether the MCA blood flow reached a systolic peak. The built-in TCD system was used to obtain the systolic velocity (Vs), diastolic velocity (Vd), and mean velocity (Vm), and these were used to determine the pulsatility index (PI) by using the following formula: PI = (Vs − Vd)/Vm for each patient.

### 2.4. Cardiac Ultrasound

The phased array fan scanning probe was chosen for bedside cardiac ultrasound, and the left ventricular ejection fraction (LVEF) was measured by using a *M*-mode ultrasound through the parasternal short-axis papillary muscle plane. The blood flow imaging through the apical five-chamber view subsequently produced the left ventricular outflow. The blood flow velocity time integral (VTI) spectrum of the left ventricular outflow tract (LOVT) was obtained by measuring the pulsed wave Doppler.

### 2.5. The Vasoactive-Inotropic Score (VIS) Calculation

The VIS was calculated after administration of dopamine (*μ*g/(kg·min)) + dobutamine (*μ*g/(kg·min)) + 10 × milrinone (*μ*g/(kg·min)) + 100 × adrenaline (*μ*g/(kg·min)) + 100 × norepinephrine (*μ*g/(kg·min)) + 10000 × vasopressin (U/(kg·min)) [17].

### 2.6. Statistical Methods

SPSS 23.0 statistical software (IBM Corp., Armonk, NY) was used for data analysis, and R4.2.0 (Lucent Technologies, Inc., Reston, VA) was used to produce figures. The Kolmogorov–Smirnov test was used to determine the normality of the data. Normally distributed data were expressed as mean ± standard deviations ($\bar{x}\pm s$), and the *t*-test was used for comparison between the two samples. Nonnormally distributed data were expressed as medians (quartiles) (*M* (QL, QU)), and the Mann–Whitney *U* test was used for comparison between the two samples. Data were expressed as relative constituent ratios (%) or rates (%). Either Pearson's chi-square test or Fisher's precision probability test was used for comparison between the two samples. The correlation analysis of binary variables was analyzed using the logistic regression model. The predicted values were calculated using the ROC curve analysis, the area under the curve (AUC), and the Youden index (YI). *P* < 0.05 was considered to be statistically significant.

## 3. Results

**Figure 1:**
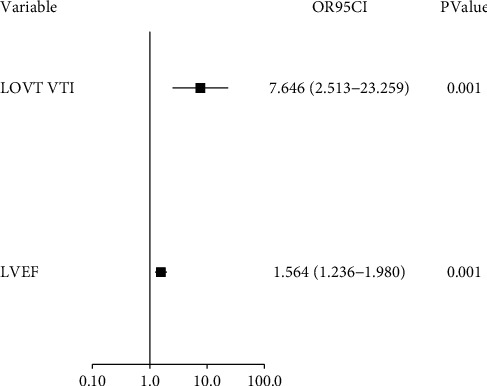
Correlation analysis of LVOT VTI, LVEF, and RMCA with the systolic peak.

LVOT VTI and LVEF are the main evaluation index of cardiac systolic function for patients. After performing correlation analysis by using the RMCA, it was found that LVOT VTI (OR = 7.646, 95% CI: 2.513∼23.259, *P*=0.001) and LVEF (OR = 1.564, 95% CI: 1.236∼1.980, *P*=0.001) were closely related to whether there was a contraction peak in the RMCA. The correlation analyses of LVOT VTI, LVEF, and the RMCA with systolic peak are shown in Figure 1.

**Figure 2:**
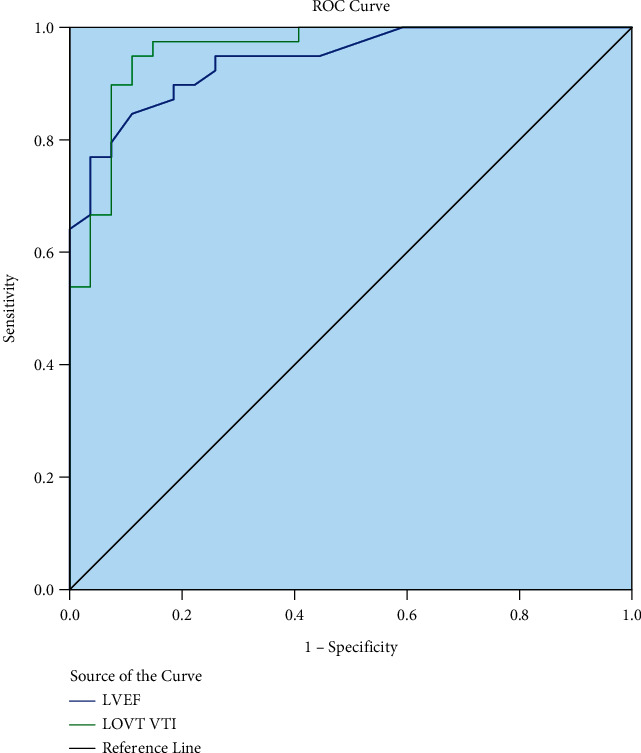
The predictive value of LVOT VTI and LVEF for the occurrence of systolic peaks in the right middle cerebral arteries.

The LVOT VTI (AUC = 0.958, 95% CI: 0.912∼1.000, *P*=0.001) and LVEF (AUC = 0.941. 95% CI: 0.890∼0.993, *P*=0.001) obtained from patients had a high predictive value to detect the systolic peak of the RMCA. The optimal critical value of LVOT VTI for predicting the appearance of the systolic peak in the RMCA was 4.17 cm (YI = 0.84), with a sensitivity and specificity of 0.95 and 0.89, respectively. The optimal critical value of LVEF for predicting the appearance of a systolic peak in the RMCA was 13.50% (YI = 0.74), with a sensitivity and specificity of 0.85 and 0.89, respectively. The predictive values obtained for LVOT VTI and LVEF for the occurrence of systolic peaks in the RMCA are shown in Table 1 and Figure 2.

During the study period, 71 patients were enrolled, but 5 patients who did not meet the inclusion criteria were excluded (1 with primary cerebral hemorrhage and 4 TCD without any acoustic windows). 66 patients with an average age 59.21 ± 9.56 were included in this study. 54.55% (*N* = 36) of the patients were male and 37 (56.06%) of them had acute myocardial infarction, which was the most common primary disease type. The patients were divided into survival and death groups according when assessed within the first 28 days after admission. A comparison of the baseline characteristics of the patients in both the groups is shown in Table 2. There were no significant differences in the comparison indicators in the two groups.

A comparison of the clinical outcomes of the survival and death groups showed that the initial LVEF (*P*=0.034), Vs (*P*=0.005), Vd (*P*=0.041), PI (*P*=0.013), initial lactate concentration (*P*=0.040), and MODS incidence rate (*P*=0.042) were statically significantly different. A comparison of the clinical data between the two groups is shown in Table 3.

**Figure 3:**
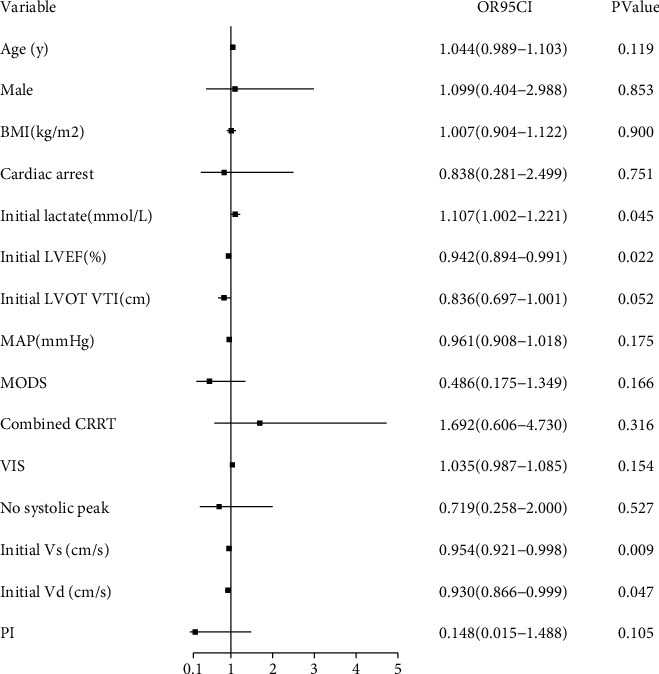
Univariate regression analysis of the variables affecting 28-day mortality of patients undergoing VA-ECMO.

Univariate regression analysis was used to determine the variables affecting the patients' 28-day mortality. Initial values obtained for the LVEF (OR = 0.942, 95% CI: 0.894∼0.991, *P*=0.022), Vs (OR = 0.954, 95% CI: 0.921∼0.998, *P*=0.009), Vd (OR = 0.930, 95% CI: 0.866∼0.999, *P*=0.047), and initial lactate concentration (OR = 1.107, 95% CI: 1.002∼1.221, *P*=0.045) were the four indicators included in the multivariate logistic regression equation (Figure 3). The results showed that the initial Vs value (OR = 0.954, 95% CI: 0.921∼0.988, *P*=0.009) was an independent risk factor that affected the patient's 28-day mortality after VA-ECMO.

## 4. Discussion

The cerebral hemodynamics of patients were found to be different under different cardiac function states. Previous studies had shown that TCD ultrasound can detect nonpulsatile cerebral blood flow in VA-ECMO patients with significantly reduced LVEF (LVEF <10%), [18, 19] and decreasing PI. As the LVEF increases, there is an accompanying increase in the systolic stroke amplitude which leads to an increase in PI [19]. Another study [20] found that TCD could only detect the systolic peak of cerebral blood flow when LVEF >20%, and the pattern of TCD changed with a change in the LVEF. However, at present, the above studies are at the observational stage.

**Figure 4:**
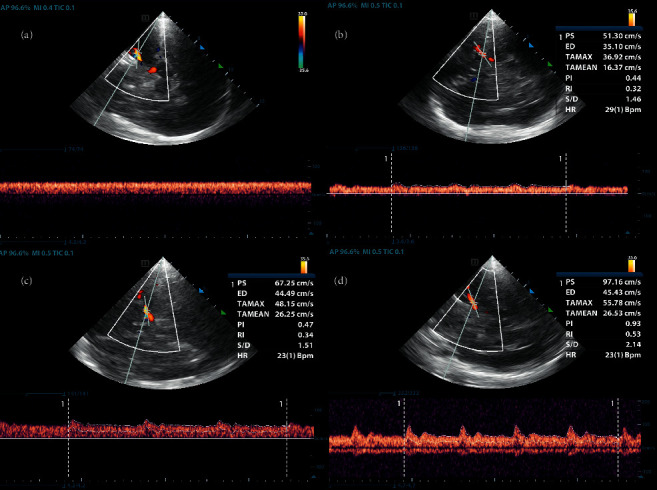
The RMCA flow waveforms that can be observed during different cardiac functional states. (a) LVEF<10%, the RMCA had an advection. (b) 10%< LVEF <20%, there was a systolic spike in the right middle cerebral artery. (c) 20%< LVEF <30%, the systolic peak of the RMCA increased further, but the PI was still below the normal value. (d) LVEF >40%, the normal blood flow pattern of the RMCA.

The results found in this study were consistent with previous studies. When the cardiac function was extremely poor, the cerebral perfusion depended on the nonpulsatile blood flow provided by VA-ECMO, and the cerebral blood flow was in a nonpulsatile state. LVOT VTI and LVEF were closely related as to whether there was a contraction peak in the RMCA. By using ROC curve calculations, it was found that a patient's LVOT VTI and LVEF had a high predictive value for the systolic peak of the RMCA. The optimal critical value of LVOT VTI for predicting the appearance of a systolic peak in the RMCA was 4.17 cm, and the optimal critical value of LVEF for predicting the appearance of the systolic peak in the RMCA was 13.50%. In some patients with certain types of cardiac contractility, a systolic peak of cerebral blood flow existed, but the PI value was usually lower than normal. As the cardiac systolic function changed, the patient's cerebral hemodynamics and PI value were also seen to change (Figure 4). This finding is consistent with several previous studies [19, 21, 22]. Kavi et al. [19] pointed out that the existence of low PI can be explained by the pulse produced by a severely weakened but mildly preserved cardiac systolic function, and such a patient should not be considered for either cerebral vasodilation or cerebral circulation arrest protocols.

Different from previous studies, we investigated the effect of cerebral hemodynamic changes on the prognosis of VA-ECMO patients. We divided our patients into two based on whether they survived up to the 28th day of admission and compared different parameters in the survival and death groups. We found that the initial LVEF, Vs，Vd, and PI values of the survival group were higher than those of the death group, while the proportion of no systolic peak was lower than that of the death group. This led to the conclusion that the cerebral blood flow state, which was closely related to cardiac function, could be used as the prognosis evaluation indicator of the patients. According to the results of univariate correlation analysis of the 28-day mortality, the initial LVEF, Vs, Vd, and lactate levels were included in the multivariate logistic regression equation. This showed that the initial Vs value was an independent risk factor that affected the prognosis of VA-ECMO patients, and the poor cerebral arterial pulsing blood flow was significantly related to the poor prognosis of patients. Notably, none of the surviving patients with advection in their initial cerebral blood flow in this study had adverse neurological outcomes. This suggests that nonpulsatile perfusion or a low PI state supported by VA-ECMO may not have a negative effect on neurological functions, while long-term effects may remain and this needs to be further investigated.

There were still some limitations in this study. First, the small sample size may have affected the test performance, and clinical subgroup analysis could not be performed. It cannot be determined whether the outcome indicators were affected by the classification of the primary disease. Second, indicators such as EEG and cerebral oxygen saturation monitoring were not included in the study to comprehensively assess the state of brain function. In future studies, we will further explore the potential correlation between changes in the cerebral blood flow patterns and neurological complications in VA-ECMO patients. We will also conduct risk predictions and stratification studies by using TCD models, which may require the support of multiple centers and a large sample size. Meanwhile, the question of how to evaluate the cerebral vascular autoregulation function and the intervention of cerebral perfusion in the TCD mode under the influence of cardiopulmonary bypass remain to be explored.

## 5. Conclusions

Patients supported by VA-ECMO may have cerebral hemodynamic changes resulting in poor cerebral arterial pulsatile blood flow and this is closely related to their poor prognosis. The TCD ultrasound provides a convenient, real-time, and noninvasive method to perform bedside dynamic monitoring of the cerebral blood flow status in VA-ECMO patients. This simple and cost-effective procedure has broad application prospects in cerebral hemodynamic monitoring, prognosis assessment, and cerebral perfusion interventions in patients undergoing VA-ECMO.

## Figures and Tables

**Table 1:** The predictive value of LVOT VTI and LVEF for the occurrence of systolic peaks in the right middle cerebral arteries.

Variable	YI	Cutoff	Sensitivity	Specificity	AUC	*P*	95% CI
LVOT VTI (cm)	0.84	4.17	0.95	0.89	0.941	0.001	0.890∼0.993
LVEF (%)	0.74	13.50	0.85	0.89	0.958	0.001	0.912∼1.000

**Table 2:** Comparison of baseline characteristics between the survival and death groups.

Variable	Survival group (*n* = 25)	Death group (*n* = 41)	Statistics	*P*
Age (*y*)	55.60 ± 11.91	60.85 ± 8.40	1.932	0.061
Male/female (*n*)	14/11	22/19	0.034	0.853
BMI (kg/m^2^)	24.23 ± 5.25	24.38 ± 4.33	-0.124	0.901
Hypertension (*n* %)	12 (48.00)	17 (17.07)	0.269	0.604
Diabetes mellitus (*n* %)	8 (32.00)	7 (21.95)	1.970	0.160
Smoking (*n* %)	7 (28.00)	11 (26.83)	0.011	0.917

Classification of primary diseases (*n* %)				
Myocardial infarction	13 (52.00)	24 (58.53)	0.269	0.604
Heart failure	9 (36.00)	12 (29.27)	0.324	0.569
Pulmonary embolism	3 (12.00)	1 (2.44)	1.097	0.295^a^
Congenital heart disease	0 (4.00)	3 (4.88)	0.601	0.438^a^
Severe sepsis	0	1 (2.44)	--	0.621^b^

*Note.* ^a^Continuous correction by the chi-square test was used. ^b^Fisher's precision probability test was used.

**Table 3:** Comparison of clinical data between the survival and death groups.

Variable	Survival group (*n* = 25)	Death group (*n* = 41)	Statistics	*P*
Cardiac arrest (*n* %)	7 (28.00)	13 (31, 70)	0.101	0.751
Initial lactate (mmol/L)	11.64 ± 4.65	14.49 ± 5.74	−2.095	0.040

Initial cardiac function				
LVEF (%)	21.10 ± 12.72	14.88 ± 7.94	2.199	0.034
LVOT VTI (cm)	6.11 ± 3.29	4.63 ± 2.58	1.914	0.064
MAP (mmHg)	69.88 ± 9.64	66.63 ± 9.08	1.376	0.174
MODS (*n* %)	5 (20.00)	18 (43.90)	3.908	0.048
Combined CRRT (*n* %)	11 (44.00)	13 (31.71)	1.014	0.314
VIS	34.44 ± 11.53	38.54 ± 10.94	−1.446	0.153

Initial RMCA flow				
No systolic peak (*n* %)	9 (36.00)	18 (43.90)	0.401	0.526
Vs (cm/s)	57.56 ± 15.63	46.45 ± 14.83	2.894	0.005
Vd (cm/s)	38.87 ± 8.30	34.90 ± 6.96	2.089	0.041
PI (IQR)	0.48 (0.37, 0.55)	0.36 (0.32, 0.42)	−2.459	0.013^a^

*Note.* IQR, interquartile range. ^a^Mann–Whitney *U* test was used.

## Data Availability

The datasets used and analyzed during the current study are available from the corresponding author upon request.
